# Effects of sugar-sweetened soda on plasma saturated and monounsaturated fatty acids in individuals with obesity: A randomized study

**DOI:** 10.3389/fnut.2022.936828

**Published:** 2022-08-31

**Authors:** Mohammed Fahad Bajahzer, Jens Meldgaard Bruun, Fredrik Rosqvist, Matti Marklund, Bjørn Richelsen, Ulf Risérus

**Affiliations:** ^1^Clinical Nutrition and Metabolism, Department of Public Health and Caring Sciences, Faculty of Medicine, Uppsala University, Uppsala, Sweden; ^2^Steno Diabetes Center Aarhus, Aarhus University Hospital, Aarhus, Denmark; ^3^Department of Clinical Medicine, Aarhus University, Aarhus, Denmark; ^4^The George Institute for Global Health, Faculty of Medicine, UNSW Sydney, Sydney, NSW, Australia; ^5^Department of Epidemiology, Johns Hopkins Bloomberg School of Public Health, Johns Hopkins University, Baltimore, MD, United States

**Keywords:** sugar-sweetened beverages, carbohydrate intake, *de novo* lipogenesis, plasma fatty acid composition, saturated, monounsaturated, liver fat, biomarkers

## Abstract

**Background:**

High carbohydrate, i.e., sugars, intake potentially drives the liver into a lipogenic state leading to elevated plasma fatty acids. Excessive intake of saturated fat and sugar-sweetened soda induces liver fat accumulation, but studying the effect of high intake from sugar-sweetened soda on the *de novo* lipogenesis (DNL) fatty acids in long-term randomized trials is lacking.

**Objective:**

To study the effect of consuming 1 L/day of sugar-sweetened soda, semi-skimmed milk (milk), aspartame-sweetened soda or water over 24 weeks on DNL-derived fatty acids (i.e., palmitate (primary outcome) and other saturated and monounsaturated fatty acids), and markers of stearoyl-CoA desaturase activity (SCD1) in plasma phospholipids (PL), cholesteryl esters (CE), and triglycerides (TG).

**Design and methods:**

A randomized parallel study was conducted simultaneously at Aarhus University Hospital and Copenhagen University, Denmark, including (*n* = 41) individuals aged 20–50 years, with BMI of 26–40 kg/m^2^, and without diabetes. The groups consisted of 9 individuals in the sugar-sweetened soda, 10 in the milk, 11 in the aspartame-sweetened soda, and 11 in the water. The change at 24 weeks was assessed and compared across the groups using ANCOVA and mixed-effects models. Correlations of fatty acid changes with liver fat accumulation (magnetic resonance imaging) were explored.

**Results:**

After 24 weeks, the groups differed in palmitate proportions in PL, oleate in CE and PL, and palmitoleate and SCD1 in all fractions (*p* < 0.05). Compared with water, the relative proportion of palmitate in PL increased by approximately 1% during both sugar-sweetened soda (*p* = 0.011) and milk (*p* = 0.006), whereas oleate and palmitoleate increased only during sugar-sweetened soda (CE 2.77%, *p* < 0.001; PL 1.51%, *p* = 0.002 and CE 1.46%, PL 0.24%, TG 1.31%, all *p* < 0.001, respectively). Liver fat accumulation correlated consistently with changes in palmitoleate, whereas correlations with palmitate and oleate were inconsistent across lipid fractions.

**Conclusions:**

Although both sugar-sweetened soda and milk increased palmitate in PL, only excess intake of sugar-sweetened soda increased palmitoleate in all lipid fractions and correlated with liver fat. In contrast, isocaloric milk intake did not increase plasma monounsaturated fatty acids.

**Clinical trial registration:**

[https://clinicaltrials.gov/ct2/show/NCT00777647], identifier [NCT00777647].

## Introduction

*De novo* lipogenesis (DNL) is the metabolic pathway by which fatty acids are endogenously synthesized, mainly from excess intake of carbohydrates. DNL is increased in nonalcoholic fatty liver disease (NAFLD) (1, 2), whereas in healthy subjects, DNL may be upregulated due to excessive energy intake composed mainly of high carbohydrate (i.e., sugars) and low-fat diets (3). When glycogen depots in the liver and muscles are saturated, glycogenesis is inhibited, whereas DNL is stimulated in the presence of insulin to utilize plasma glucose to synthesize palmitate (16:0), the primary DNL product (4). Synthesized 16:0 can further be elongated into other saturated fatty acids (SFA), e.g., stearate (18:0), and/or desaturated by stearoyl-CoA desaturase (SCD1) to monounsaturated fatty acids (MUFA), e.g., palmitoleate (16:1n7) (5). This notion could partly explain the weak association between circulating even-chain SFA and dietary SFA, as carbohydrates might at very high intakes induce *de novo* synthesis of even-chain SFA and potentially increase these SFA in circulating lipids (6). Therefore, plasma 16:0 and some MUFA might reflect carbohydrate intake in certain circumstances, especially in the cases of excessive intake of sugars, as they are more lipogenic than complex carbohydrates (7). While higher circulating 16:0 and SCD1-derived MUFA levels may be potential biomarkers of excessive sugar intake (8), in addition to reflecting increased saturated fat intake (9), more studies are needed to assess the generalizability to different dietary conditions and in various populations.

Since increased 16:0 in plasma is consistently linked to an increased risk of cardiometabolic diseases (10–12), it is important to understand different dietary contributors to plasma 16:0. Previous interventional studies have had short duration (only days or a few weeks) and with different doses and types of carbohydrates and fat have yielded inconsistent results (13–16). In addition, the reported data on the effect of intake of sugar-sweetened beverages on the DNL have commonly done so in populations with a dietary pattern that is characterized by excess intake of added sugars, especially fructose (17–20). Another distinct feature of the aforementioned populations is the commercially available sugar-sweetened beverages that are typically sweetened with high-fructose corn syrup, while, on the contrary, such beverages are sweetened with sucrose, for instance, in Europe (21). Thus, whereas fructose intake has been of great interest, sugar intake *per se* is also of importance. In several, but not all (22), shorter-term studies, diets rich in sugar and/or sugar-sweetened beverages containing fructose were found to induce DNL in most (17, 19, 23–26), and the potential links to liver fat accumulation are unclear. Therefore, it is of interest to investigate excessive sugar intake over an extended period to understand the effects on plasma SFA and MUFA and to examine the possible link to liver fat accumulation. RCTs with long duration using sugar-sweetened soda (SS), in addition to previous short-term studies, are needed to understand responses in plasma 16:0 after excessive sugar intake from commonly consumed beverages. Such data could also be useful in interpreting fatty acid data in epidemiological and interventional studies. The aim of this secondary analysis study was to investigate the effect of high intake of SS on plasma SFA and MUFA in different lipid fractions, as compared to daily consumption of aspartame-sweetened soda (AS), semi-skimmed milk, and water over 24 weeks. We also investigated the correlation between changes in plasma fatty acids and changes in liver fat content.

## Materials and methods

### Study design and intervention

The study population and protocol have been described in detail previously (27). In brief, the study was undertaken simultaneously at Aarhus University Hospital and the Department of Nutrition, Exercise, and Sports, Copenhagen University, Denmark. Participants without diabetes (*n* = 47) with a BMI range of 26–40 kg/m^2^ and age between 20 and 50 years were included in the study (Table 1). Exclusion criteria included diagnosis with diabetes, hypertension, pregnancy, lactose intolerance, or phenylketonuria. Subjects were also excluded if they were smoking, breastfeeding, exercising more than 10 h/week, or using medications that affect body weight, blood glucose, or lipids. Body implants, i.e., made of metal, were an additional cause for exclusion due to the conflict with magnetic resonance imaging (MRI) scans. All participants provided their written informed consent. The Ethical Committee of Middle Jutland, Denmark, approved the study protocol, and the study was conducted according to the Declaration of Helsinki.

**TABLE 1:** Baseline characteristics.

	Sugar-sweetened soda	Semi-skimmed milk	Aspartame-sweetened soda	Water
Gender (*n*) Men | Women	6 | 4	3 | 9	3 | 9	5 | 8
Age	39 ± 6	38 ± 9	39 ± 8	39 ± 8
BMI	31.3 ± 2.9	31.9 ± 2.8	32.8 ± 3.8	32.2 ± 4.6
Fasting plasma insulin (pmol/L)	54.3 ± 26.7	92.6 ± 74.9	79.0 ± 30.0	80.6 ± 58.0
Fasting plasma glucose (mmol/L)	5.4 ± 0.6	5.4 ± 0.8	5.5 ± 0.5	5.3 ± 0.6
Triglycerides (mmol/L)	1.1 ± 0.3	1.7 ± 0.9	1.7 ± 0.6	1.7 ± 0.8
Total cholesterol (mmol/L)	4.9 ± 1.0	5.3 ± 1.0	5.2 ± 0.7	5.2 ± 0.8
HDL cholesterol (mmol/L)	1.2 ± 0.2	1.1 ± 0.2	1.2 ± 0.3	1.1 ± 0.3

Data values are presented as mean ± SEM.

**FIGURE 1:**
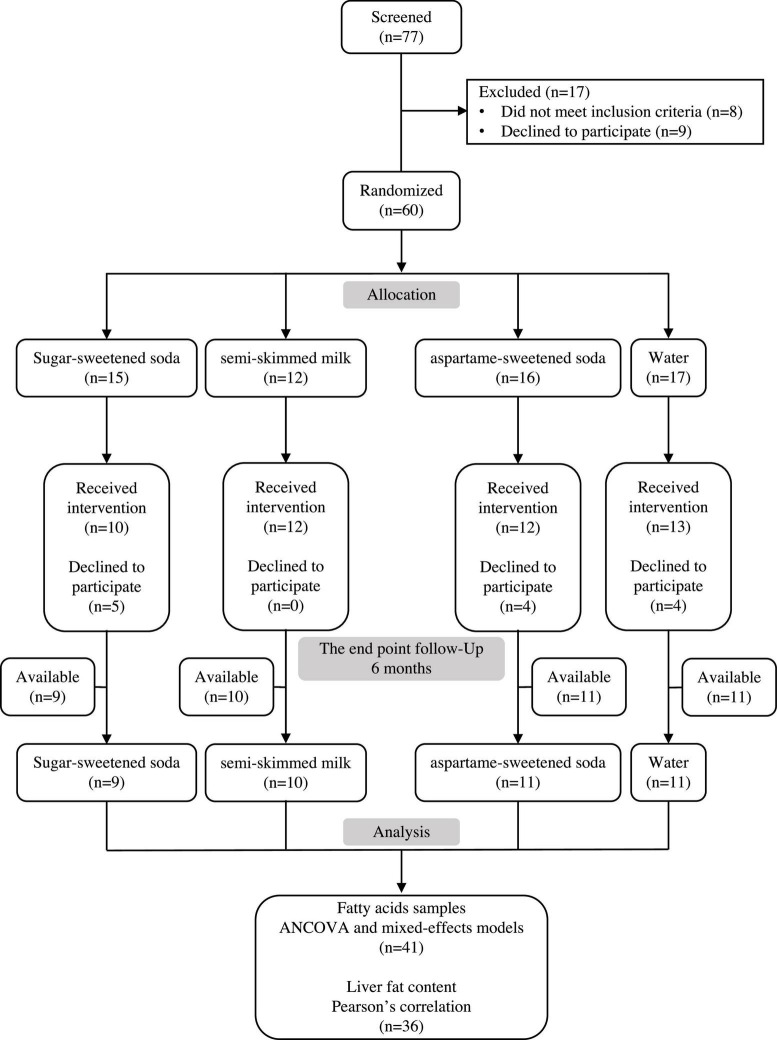
The consort flow diagram. Seventy-seven subjects were screened for eligibility to enroll in the study and 60 subjects were randomized.

The study design consisted of four parallel treatment groups (Figure 1). In total, 60 subjects were randomized to the test beverages, 13 declined to participate after the allocations, leaving 47 subjects for fatty acid analyses (Figure 1). The participants were randomly assigned to consume one of the four test beverages on a daily basis for 24 weeks. The test beverages included (1 L/day) SS, semi-skimmed milk, AS, or water (Table 2). All the test beverages were provided free of charge two or three times per month by the research centers. The subjects were asked to return the empty containers to the research center every 3–4 weeks to monitor and motivate compliance. Furthermore, compliance checks were performed every 6 weeks. Subjects who were randomly assigned to consume soft drinks were provided with toothpaste, instructed about dental hygiene, and examined by a dentist at baseline and every 1.5 months throughout the intervention to check for caries or acid erosion of the enamel. None of the subjects developed these problems.

**TABLE 2:** Composition of the test beverages.

	Sugar-sweetened soda	Semi-skimmed milk	Aspartame-sweetened soda	Water
Volume (ml/day)	1,000	1,000	1,000	1,000
**Carbohydrate (g/1,000 ml)**				
Sucrose	106	0	0	0
Lactose	0	47	0	0
Protein (g/1,000 ml)	0	34	<1	0
Fat (g/1,000 ml)	0	15	0	0
Energy (kcal/1,000 ml)	450.0	474.9	3.6	0

### Measurements

#### The composition of plasma fatty acid

Blood samples were collected in the morning after an overnight fasting. Subjects were asked to refrain from any occasional medicine, alcohol, or vigorous exercise for 24 h before the visit. Samples of blood plasma were aliquoted into several tubes and stored at –80°C. Due to the prior use of the samples in previous analyses, only 41 blood samples were available at both the baseline and end-point (24 weeks) (Figure 1). The samples were transported to the Unit of Clinical Nutrition and Metabolism, the Department of Public Health and Caring Sciences, Uppsala University, Sweden to analyze the composition of fatty acids in blood plasma. Plasma lipids were extracted using Folch’s extraction method (chloroform/methanol) (28). Thereafter, lipid fractions were separated using thin-layer chromatography (TLC) to obtain cholesteryl ester (CE), phospholipid (PL), and triglyceride (TG) fractions from the plasma. The lipid fractions were then individually extracted from the TLC plates and prepared further to assess the composition of fatty acid using the gas–liquid chromatography technique as previously described (29). Fatty acids were expressed accordingly as percentages of total fatty acids in the investigated plasma lipids. The activity of the SCD1 enzyme was estimated using a 16:1n7 to 16:0 ratio. The primary outcome was 16:0, and the secondary outcomes included myristate (14:0), the SCD1 products (16:1n7) and oleate (18:1n9), and its latter precursor (18:0).

#### Assessment of liver fat content

Intrahepatic fat content was assessed with MRI techniques using a Signa Excite 1.5 Tesla Twin-Speed scanner (GE Medical Systems, Milwaukee, WI, United States), as previously described in detail (27). Due to some difficulties during procedures of the MRI scan, as reported during the primary investigations, MRI data on liver fat was only available in 36 subjects.

### Statistical analysis

Fatty acids distributions were evaluated, and 18:0 values were log-transformed to approximate normality before statistical analyses due to a skewed distribution. Fatty acids at the end of the intervention (i.e., after 24 weeks) were utilized to examine the difference between the groups. A completer (*n* = 41) analysis of covariance (ANCOVA) was computed, including group, baseline values of fatty acids, sex, body weight at the end of the study, and age as covariates. Baseline and endpoint fatty acids data were utilized in mixed-effects models with water as the reference treatment, to assess differences in changes between the reference and each of the alternative treatments. The mixed effects model included the fatty acid of interest as the dependent variable (outcome); individual as a random intercept; group, time, body weight, sex, and age as fixed effects, and an interaction term of group and time. Exploratory correlations between the change in log-transformed plasma fatty acid levels and the change in liver fat content during 24 weeks of the intervention were assessed using Pearson’s correlation coefficients (for the whole population, *n* = 36). All analyses were performed using Stata version 15.1 (StataCorp LCC); two-sided *p*-values < 0.05 were considered statistically significant.

## Results

### Participant characteristics

The baseline characteristics of the participants are shown in Table 1. The groups were matched for age and BMI, but there was an unequal distribution of sex between the groups. A total of 13 women dropped out after randomization; of which 4 were assigned to water, 5 to SS, and 4 to AS. A total of 47 subjects (30 women and 17 men) completed the study. Due to the prior use of the samples for analyses in previous publications, some blood samples [1 SS, 2 milk, 1 AS, and 2 water (Figure 1)] were insufficient at both the baseline and after 24 weeks, leaving 41 blood samples that have been used in the analysis of this study. At baseline, SS intake was not different between the 4 groups, and the mean intake in these subjects was 184 ml/day.

### Treatment effects on fatty acids in the *de novo* lipogenesis pathway

**FIGURE 2:**
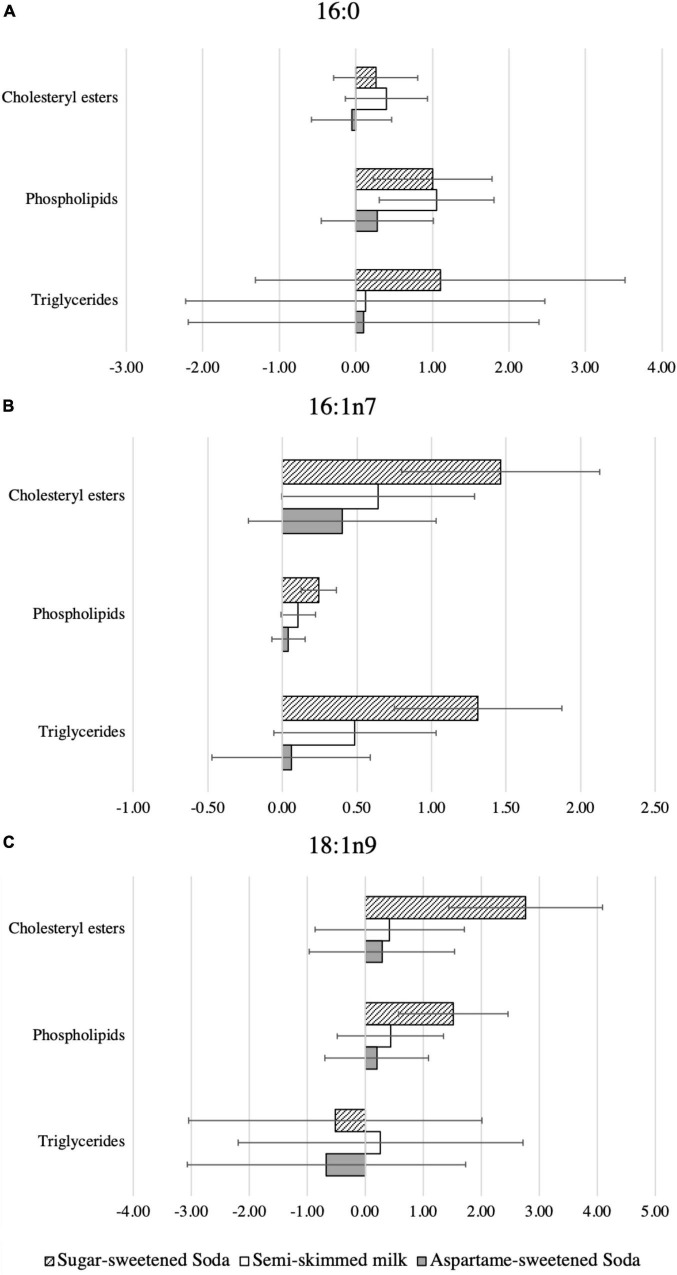
Data are differences in the relative changes of plasma fatty acids comparing water with each of the test beverages. Percentages of the relative changes as indicated by the mixed effects model, with each individual fatty acid as the dependent variable; individual as a random intercept; group, time, weight, sex, and age as fixed effects; and an interaction term of group and time. **(A)** Palmitate (16:0), **(B)** palmitoleate (16:1n7), and **(C)** oleate (18:1n9). The columns in the charts exhibit the mean values of relative change (the baseline values were subtracted from the 24 weeks values) in the levels of each one of the included fatty acids in each plasma lipid fraction for the included test beverage, in comparison to water. The distinct columns exhibit three test beverages; the diagonal pattern columns represent sugar-sweetened soda, the white columns represent semi-skimmed milk, and the gray columns represent aspartame-sweetened soda.

After adjustments for baseline levels of the investigated fatty acids, sex, age, and body weight, the proportions of 16:0 in PL, but not in CE or TG, differed between groups (ANCOVA *p* = 0.026) at the end of the treatment (Table 3 and Supplementary Table 1). Compared with water, 16:0 increased in PL, but not in CE or TG, during both SS (mean difference:1.0%; 95% CI:0.2,1.8; *p* = 0.011) and milk (1.1%; 0.3,1.8; *p* = 0.006) (Figure 2 and Supplementary Table 2).

**TABLE 3:** Changes in plasma of fatty acids in different lipid fractions.

Fatty acid	Fraction	Sugar-sweetened soda (*n* = 9)		Semi-skimmed milk (*n* = 10)		Aspartame-sweetened soda (*n* = 11)		Water (*n* = 11)		*p*
					
		Baseline	Change	Baseline	Change	Baseline	Change	Baseline	Change	
14:0	CE	0.52 (0.45, 0.58)	0.19 (0.07, 0.31)	0.69 (0.57, 0.81)	0.16 (0.01, 0.30)	0.69 (0.63, 0.75)	–0.02 (–0.10, 0.06)	0.81 (0.69, 0.93)	–0.07 (–0.17, 0.04)	0.030
	PL	0.30 (0.24, 0.35)	0.07 (–0.01, 0.16)	0.32 (0.27, 0.37)	0.08 (–0.02, 0.19)	0.36 (0.32, 0.40)	0.00 (–0.04, 0.03)	0.37 (0.32, 0.42)	–0.01 (–0.05, 0.03)	0.443
	TG	1.54 (1.07, 2.01)	0.59 (–0.18, 1.36)	2.13 (1.56, 2.69)	0.48 (–0.21, 1.17)	2.26 (1.89, 2.62)	0.07 (–0.38, 0.52)	2.21 (1.84, 2.58)	0.17 (–0.17, 0.51)	0.629
16:0	CE	11.72 (11.19, 12.25)	–0.23 (–0.87, 0.42)	11.97 (11.47, 12.47)	–0.08 (–0.58, 0.42)	12.01 (11.42, 12.60)	–0.54 (–0.92, -0.16)	11.92 (11.55, 12.29)	–0.49 (–0.85, -0.12)	0.297
	PL	29.13 (28.16, 30.10)	0.22 (–0.73, 1.17)	29.36 (28.24, 30.48)	0.28 (–0.50, 1.05)	29.56 (28.52, 30.60)	–0.50 (–1.01, 0.01)	29.53 (28.70, 30.36)	–0.78 (–1.12, -0.44)	0.026
	TG	26.25 (24.64, 27.87)	1.18 (–1.19, 3.55)	30.10 (27.44, 32.76)	0.21 (–1.48, 1.90)	29.45 (27.76, 31.14)	0.17 (–2.16, 2.50)	28.41 (26.48, 30.35)	0.08 (–1.72, 1.88)	0.781
16:1n7	CE	2.74 (1.99, 3.49)	0.92 (0.21, 1.62)	3.66 (2.49, 4.82)	0.10 (–0.63, 0.83)	3.56 (2.91, 4.21)	–0.15 (–0.51, 0.22)	3.61 (2.92, 4.29)	–0.55 (–1.03, -0.06)	0.012
	PL	0.47 (0.38, 0.56)	0.18 (0.05, 0.30)	0.59 (0.41, 0.77)	0.04 (–0.07, 0.15)	0.59 (0.49, 0.69)	–0.03 (–0.09, 0.04)	0.59 (0.47, 0.70)	–0.07 (–0.17, 0.04)	0.016
	TG	3.71 (3.34, 4.08)	1.06 (0.48, 1.63)	4.43 (3.42, 5.44)	0.23 (–0.44, 0.89)	4.50 (3.85, 5.14)	–0.20 (–0.46, 0.06)	4.45 (3.76, 5.13)	–0.26 (–0.62, 0.10)	0.001
18:0	CE	0.94 (0.74, 1.14)	–0.14 (–0.29, 0.01)	0.88 (0.79, 0.98)	–0.01 (–0.07, 0.06)	0.92 (0.75, 1.09)	–0.12 (–0.29, 0.06)	0.85 (0.76, 0.93)	0.01 (–0.07, 0.08)	0.082
	PL	14.22 (13.75, 14.68)	0.13 (–0.48, 0.73)	14.67 (13.86, 15.48)	–0.17 (–0.56, 0.23)	14.15 (13.07, 15.23)	0.60 (0.09, 1.10)	14.24 (13.51, 14.96)	0.62 (0.26, 0.98)	0.055
	TG	3.73 (3.10, 4.35)	–0.10 (–0.94, 0.74)	3.82 (3.33, 4.30)	0.08 (–0.27, 0.44)	4.03 (3.40, 4.66)	0.06 (–0.59, 0.71)	3.72 (3.30, 4.13)	0.47 (0.02, 0.91)	0.326
18:1n9	CE	21.73 (20.28, 23.18)	1.91 (0.24, 3.58)	22.07 (21.04, 23.10)	–0.44 (–1.68, 0.80)	21.45 (20.36, 22.54)	–0.57 (–1.50, 0.36)	21.51 (20.75, 22.27)	–0.86 (–1.49, -0.22)	0.001
	PL	11.01 (10.56, 11.46)	1.23 (0.26, 2.20)	11.00 (10.27, 11.73)	0.15 (–0.69, 0.98)	10.85 (10.35, 11.35)	–0.09 (–0.80, 0.62)	11.04 (10.59, 11.49)	–0.29 (–0.99, 0.42)	0.019
	TG	45.57 (43.29, 47.85)	–0.87 (–3.62, 1.88)	42.11 (39.52, 44.69)	–0.10 (–1.50, 1.31)	42.85 (41.14, 44.57)	–1.03 (–3.44, 1.38)	43.18 (41.29, 45.07)	–0.36 (–2.15, 1.43)	0.600
SCD1	CE	0.23 (0.17, 0.29)	0.08 (0.03, 0.14)	0.30 (0.21, 0.39)	0.01 (–0.05, 0.07)	0.30 (0.25, 0.34)	0.00 (–0.03, 0.03)	0.30 (0.25, 0.35)	–0.04 (–0.07, 0.00)	0.009
	PL	0.02 (0.01, 0.02)	0.01 (0.00, 0.01)	0.02 (0.01, 0.03)	0.00 (0.00, 0.00)	0.02 (0.02, 0.02)	0.00 (0.00, 0.00)	0.02 (0.02, 0.02)	0.00 (–0.01, 0.00)	0.023
	TG	0.14 (0.13, 0.15)	0.03 (0.01, 0.05)	0.15 (0.12, 0.18)	0.01 (–0.01, 0.02)	0.15 (0.13, 0.17)	0.01 (–0.02, 0.01)	0.16 (0.14, 0.17)	–0.01 (0.02, 0.00)	0.000

Included in this table are the baseline proportions of fatty acids in plasma lipid fractions and the change after 24 weeks of intervention. Values are means (95% confidence interval). The ANCOVA p-value indicates the difference between the groups.

The 16:1n7 and SCD1 activity index differed between groups at the end of the treatment in all plasma lipid fractions (ANCOVA *p* ≤ 0.023) (Table 3). Compared with water, SS intake, but not milk or AS intake, increased 16:1n7 consistently across lipid fractions (Supplementary Table 2). Similarly, compared with water, SS intake increased SCD1 activity across lipid fractions, milk increased SCD1 in TG only (0.02%; 0.00, 0.03; *p* = 0.049), and no clear effect on SCD1 activity index in CE, PL, or TG lipid fractions was caused by AS intake (Supplementary Table 2).

Levels of 14:0 differed between groups in CE (ANCOVA *p* = 0.03) and compared to water, the 14:0 levels increased during both SS (0.26%; 0.12, 0.39; *p* < 0.001) and milk (0.22%; 0.09, 0.36; *p* = 0.001) (Table 3 and Supplementary Table 2). Levels of 14:0 did not differ overall greatly between the groups in the PL and TG (ANCOVA *p* = 0.443, and *p* = 0.629, respectively) (Table 3), although when compared to water, modestly higher 14:0 levels in CE were observed for the SS group (0.26%; 0.12, 0.39; *p* < 0.001) and the milk group (0.22%; 0.09, 0.36; *p* = 0.001) (Supplementary Table 2).

The increase in 18:1n9 levels in PL and CE was similar to those of 16:1n7 and SCD1 activity, although no clear treatment effect was observed in TG (Figure 2 and Supplementary Table 2).

No consistent treatment effects were observed for 18:0, although PL 18:0 decreased (–0.05%, –0.09, –0.02; *p* = 0.005) after milk (compared to water). Differences between groups and estimated treatment effects on other measured fatty acids are available in the online Supplementary material (Supplementary Tables 1, 2).

### Exploratory correlations between fatty acids and liver fat content

**FIGURE 3:**
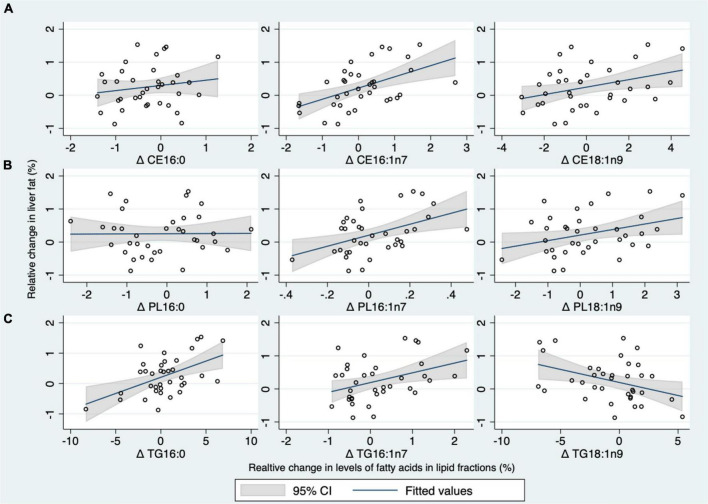
**(A–C)** Scatterplots of correlation of the relative changes (i.e., percentage points) in plasma palmitate (16:0), palmitoleate (16:1n7), oleate (18:1n9) in phospholipids (PL), cholesteryl esters (CE), and triglycerides (TG) lipid fractions with the relative changes in liver fat content after 24 weeks intervention in the whole population. The correlation of relative changes in plasma (16:0, 16:1n7, and 18:1n9) in the CE fraction with relative changes in liver fat content **(A)**, The correlation of relative changes in plasma (16:0, 16:1n7, and 18:1n9) in the PL fraction with relative changes in liver fat content **(B)**, and the correlation of relative changes in plasma (16:0, 16:1n7, and 18:1n9) in the TG fraction with relative changes in liver fat content **(C)**.

Change in liver fat content was correlated with change in 16:0 in TG (*r* = 0.50, *p* = 0.002), but not in other fractions (*r* ≤ 0.16, *p* ≥ 0.34) (Figure 3 and Table 4). The change in 14:0 correlated with liver fat accumulation in CE only (Table 4), while there was no clear correlation between changes in liver fat and the change of 18:0 in any fraction. Liver fat accumulation was correlated with changes in 16:1n7 in all fractions (*r* ≥ 0.39, *p* ≤ 0.02) (Figure 3 and Table 4), whereas changes in SCD1 were correlated in CE and PL (*r* ≥ 0.47, *p* ≤ 0.004) but not in TG (*r* = 0.14, *p* = 0.43). Liver fat accumulation was correlated directly with increases in 18:1n9 in CE and PL (*r* ≥ 0.34, *p* ≤ 0.04) but surprisingly correlated inversely with the same fatty acid measured in TG (*r* = –0.38, *p* = 0.02). Correlations between changes in liver fat and changes in other measured fatty acids are presented in the online Supplementary material (Supplementary Table 3).

**TABLE 4:** Correlations between the change of liver fat content and the change of plasma fatty acid composition in all participants.

	*r*	*p*-value
**Cholesteryl esters**		
14:0	0.352	0.035
6:0	0.163	0.342
16:1n7	0.511	0.001
18:0	–0.135	0.434
18:1n9	0.352	0.035
SCD1	0.511	0.001
**Phospholipids**		
14:0	0.243	0.154
16:0	0.008	0.961
16:1n7	0.449	0.006
18:0	0.087	0.613
18:1n9	0.339	0.043
SCD1	0.467	0.004
**Triglycerides**		
14:0	0.296	0.080
16:0	0.504	0.002
16:1n7	0.387	0.020
18:0	0.242	0.154
18:1n9	–0.383	0.021
SCD1	0.135	0.434

Data are Pearson’s correlation (r) of the change in liver fat content with the change in plasma fatty acid composition from baseline to the end of the intervention in all the participants (n = 36). Log-transformed data of liver fat content was utilized to compute Pearson’s correlation. p-value exhibits the significance levels.

## Discussion

In this study, intake of SS over 24 weeks increased plasma 16:0 in PL but not in CE or TG fractions. A similar increase was also evident during the intake of milk in PL compared with water. Levels of 14:0 increased in a comparable manner during intake of SS and milk in CE. Levels of 16:1n7 and SCD1 increased consistently during intake of SS in the three fractions, and 18:1n9 increased in a comparable manner in CE and PL. Thus, 16:1n7 seemed to be the most responsive fatty acid with regard to excessive intake of SS, and possibly a better marker than the direct DNL product 16:0. In addition, we demonstrate direct associations between the changes in 16:0 and 16:1n7 and liver fat accumulation, with consistent associations especially observed for 16:1n7 in all lipid fractions.

We have previously shown from this study that the relative change in liver fat was markedly higher after the daily intake of 1 L of SS for 24 weeks than after the daily intake of 1 L of isocaloric milk, noncaloric AS, or water (27). The change in 16:0 in the TG fraction was positively correlated with the change in liver fat. Levels of 16:1n7, and to some extent also 18:1n9, were, however, consistently positively correlated with liver fat accumulation in all lipid fractions, most evidently in CE.

It is noteworthy that isocaloric intake of milk did not induce any changes in 16:1n7 or 18:1n9, and only in 16:0 in the PL fraction. Plasma 16:0 PL was also reported to change in studies investigating distinct carbohydrate diets (controlled feeding) that showed a PL-specific change by Volk et al. but not in CE and TG fractions (14), but the change of 16:0 was shown in both PL and TG by Hyde et al. (30), and in both PL and CE by Raatz et al. (31) and King et al. (32), after higher intakes of carbohydrates, respectively. Therefore, the relevance of a PL-specific effect is unclear and not consistent among studies. However, it is possible that the higher relative abundance of 16:0 in the PL fraction, as compared with the other fractions and in CE in particular, might partly explain the current significant increase of 16:0 in PL vs. the other lipid fractions. Although SS has more than twice the carbohydrate content of milk, the total increase in carbohydrates from lactose (47 g, 188 kcal) from 1 L of milk intake per day must be regarded as a significant dietary contribution. Thus, high consumption of milk for 24 weeks does not appear to have any major effects on the fatty acid composition of the proposed lipogenic markers, except in PL 16:0. Whether this lack of effect may reflect, or possibly even explain, the lack of liver fat increase after milk intake is unclear, but an interesting possibility that requires further investigation.

We are not aware of any studies that have investigated the effect of sugar-sweetened cola or other SS on plasma fatty acid composition in different lipid fractions. In this study, we focused on the proposed lipogenic fatty acid markers that are directly (16:0) or indirectly (16:1n7, 18:1n9) involved in the lipogenic pathway. Some studies have investigated the DNL effects of increased intake of carbohydrates, either in place of fat, or as an addition to the habitual diet (17, 19, 23–26), but no studies have examined the effects of SS per se on longitudinal lipogenic fatty acid markers, i.e., in CE and PL. However, Luukkonen et al. recently showed that overfeeding (excess 1,000 kcal/day) for 3 weeks with a high-carbohydrate diet consisting of 100% simple sugars, including ∼4 dl of sugar-sweetened beverage and ∼3 dl daily of orange juice, significantly increased 16:0, 16:1n7 and 14:0 in plasma VLDL-TG, without change in 18:0 and 18:1n9 (23). Thus, although the shorter time period, and pronounced overfeeding conditions, those results are partly in line with the current findings. Of clear interest from the Luukkonen et al.’s study is the finding that isocaloric overfeeding with saturated fat (including 16:0) appeared to cause a higher increase of 16:0 in plasma VLDL-TG and a more robust increase in other SFA, including 14:0 and 18:0, compared to overfeeding with simple sugars, despite hepatic DNL being upregulated during overfeeding with sugars (23). However, this may not be surprising considering that 16:0 levels in the diet were increased, 16:0 appears to be tightly regulated and does not always increase in a pronounced fashion despite marked changes in dietary intake (33). However, other studies reporting effects of sugar-rich diets and/or sugar-sweetened beverages on lipogenic fatty acid markers have done so in VLDL-TG only, which may not be informative for interpreting results from large cohorts typically assessing fatty acids in PL and CE. Our results, reporting effects on PL and CE as well as total plasma TG, may be more informative for interpreting results from observational studies, but caution is advised when comparing with studies reporting only on VLDL-TG.

Our finding that a change in 16:1n7 in all lipid fractions, potentially reflecting increased SCD1 activity, was consistently associated with the change in liver fat content is in line with previous reports from both isocaloric and hyper-caloric trials studies in individuals fed with high amounts of saturated fat (34–36).

The randomized controlled trial design over a relatively long period of time is the main strength of this study. The equal distribution of the metabolic baseline characteristics, utilizing objective measurement to assess the composition of fatty acids, and the regularly checked compliance were other strengths of this study. Furthermore, examining the investigated change in the composition of fatty acid in the lipid fractions with the change in liver fat was an additional strength of this study. One limitation of this study was the relatively small sample size that limited the statistical power of the analyses. Although the study groups were randomized, the unknown variance in physical activity might not have been evenly distributed between the groups due to their small size. Furthermore, it should be considered that conducting multiple statistical tests without correction increases the risks of chance findings. Another possible limitation was measuring SCD1 activity indirectly using fatty acid ratios (16:1n7/16:0) in the lipid fractions. Further, due to the nature of the study, it could not be blinded that might have influenced the subjects into behavior that undermined the effects of the intervention, especially in the energy-containing test beverages.

## Conclusion

This 24-week randomized study showed that excessive intake, well above the average intake in this Scandinavian population, from both of the energy-containing beverages (SS and milk), significantly increased 16:0 in plasma PL but not in CE and TG fractions. Notably, 16:1n7 increased with greater magnitude during SS in all lipid fractions and was consistently associated with liver fat accumulation, possibly reflecting upregulated DNL and SCD1 activity in this overweight population. Thus, PL might be the most sensitive lipid fraction to capture changes in 16:0, whereas changes in 16:1n7 in response to high SS were reflected also in other lipid fractions. The isocaloric intake of milk showed a similar effect on 16:0 as SS, although milk did not induce such consistent changes in monounsaturated fatty acids, findings that are in line with the neutral effect of milk on liver fat content (27).

## Data availability statement

The original contributions presented in this study are included in the article/Supplementary material, further inquiries can be directed to the corresponding author.

## Ethics statement

The studies involving human participants were reviewed and approved by the Ethics Committee of Middle Jutland, Denmark. The patients/participants provided their written informed consent to participate in this study.

## Author contributions

UR and BR designed the research. MB, JB, FR, and MM conducted the research. MB and MM analyzed the data. MB wrote the first draft. UR had primary responsibility of the final content and conceived the study concept. All authors reviewed, provided intellectual input, revised, and approved the final manuscript.

## Abbreviations

DNL: *De novo* lipogenesis
NAFLD: non-alcoholic fatty liver disease
SCD1: stearoyl-CoA desaturase
SFA: saturated fatty acids
MUFA: monounsaturated fatty acids
SS: Sugar-sweetened soda
AS: aspartame-sweetened soda
MRI: magnetic resonance imaging
TLC: thin-layer chromatography
CE: cholesteryl esters
PL: phospholipids
TG: triglycerides
14:0: myristate
16:0: palmitate
16:1n7: palmitoleate
18:0: stearate
18:1n9: oleate.
