# Supplementary material for: Effects of sugar-sweetened soda on plasma saturated and monounsaturated fatty acids in individuals with obesity: A randomized study
Source: Front Nutr. 2022 Aug 31;9:936828. doi: 10.3389/fnut.2022.936828 (PMC9470948; doi:10.3389/fnut.2022.936828)
Supplement: Supplementary file 1 [file Data_Sheet_1.pdf]

**Supplemental Table 1. Differences between groups in plasma fatty acid composition after 24 weeks**

| Fatty acid | Fraction | Crude-*p* | Adjusted-*p* |
|---|---|---|---|
| 14:0 | CE | 0.036 | 0.030 |
| 14:0 | PL | 0.424 | 0.443 |
| 14:0 | TG | 0.636 | 0.629 |
| 15:0 | CE | 0.092 | 0.074 |
| 15:0 | PL | 0.255 | 0.148 |
| 16:0 | CE | 0.269 | 0.297 |
| 16:0 | PL | 0.032 | 0.026 |
| 16:0 | TG | 0.850 | 0.781 |
| 16:1n7 | CE | 0.010 | 0.012 |
| 16:1n7 | PL | 0.012 | 0.016 |
| 16:1n7 | TG | 0.002 | 0.001 |
| 17:0 | PL | 0.920 | 0.759 |
| 18:0 | CE | 0.130 | 0.082 |
| 18:0 | PL | 0.039 | 0.055 |
| 18:0 | TG | 0.394 | 0.326 |
| 18:1n9 | CE | 0.000 | 0.001 |
| 18:1n9 | PL | 0.007 | 0.019 |
| 18:1n9 | TG | 0.532 | 0.600 |
| 18:2n6 | CE | 0.004 | 0.005 |
| 18:2n6 | PL | 0.010 | 0.004 |
| 18:2n6 | TG | 0.519 | 0.478 |
| 18:3n6 | CE | 0.329 | 0.356 |
| 18:3n6 | PL | 0.698 | 0.725 |
| 18:3n6 | TG | 0.885 | 0.794 |
| 18:3n3 | CE | 0.702 | 0.427 |
| 18:3n3 | PL | 0.929 | 0.806 |
| 18:3n3 | TG | 0.939 | 0.954 |

| | | | |
|---|---|---|---|
| 20:0 | PL | 0.403 | 0.469 |
| 20:3n6 | CE | 0.088 | 0.097 |
| 20:3n6 | PL | 0.196 | 0.265 |
| 20:3n6 | TG | 0.533 | 0.418 |
| 20:4n6 | CE | 0.890 | 0.843 |
| 20:4n6 | PL | 0.883 | 0.879 |
| 20:4n6 | TG | 0.050 | 0.057 |
| 20:5n3 | CE | 0.274 | 0.275 |
| 20:5n3 | PL | 0.526 | 0.584 |
| 20:5n3 | TG | 0.467 | 0.500 |
| 22:0 | PL | 0.113 | 0.135 |
| 22:5n3 | PL | 0.066 | 0.096 |
| 22:5n3 | TG | 0.469 | 0.341 |
| 22:6n3 | CE | 0.493 | 0.662 |
| 22:6n3 | PL | 0.304 | 0.504 |
| 22:6n3 | TG | 0.500 | 0.602 |
| 24:0 | PL | 0.190 | 0.151 |
| SCD1 | CE | 0.008 | 0.009 |
| SCD1 | PL | 0.017 | 0.023 |
| SCD1 | TG | 0.000 | 0.000 |

Analysis of covariance (ANCOVA) in levels of plasma fatty acids between the test beverages at the end of the intervention (24 weeks). Crude-*p* column indicates *p*-values of ANCOVA including group and fatty acid value at baseline as independent variables, adjusted *p* column indicates *p*-values of ANCOVA including group, fatty acid value at baseline, sex, body weight at the end of the study, and age as independent variables.

**Supplemental Table 2. Changes in fatty acid composition in plasma lipid fractions after 3 test beverages compared with water**

| Fatty acid | Fraction | Sugar-sweetened Soda | *p*-value | Semi-skimmed Milk | *p*-value | Aspartame-sweetened Soda | *p*-value |
|---|---|---|---|---|---|---|---|
| 14:0 | CE | 0.26 (0.12, 0.39) | 0.000 | 0.22 (0.09, 0.36) | 0.001 | 0.04 (-0.09, 0.18) | 0.511 |
| 14:0 | PL | 0.08 (-0.00, 0.16) | 0.057 | 0.09 (0.01, 0.17) | 0.034 | 0.00 (-0.08, 0.08) | 0.974 |
| 14:0 | TG | 0.42 (-0.25, 1.08) | 0.222 | 0.31 (-0.34, 0.96) | 0.358 | -0.10 (-0.74, 0.53) | 0.761 |
| 15:0 | CE | 0.02 (0.00, 0.04) | 0.117 | 0.03 (0.01, 0.05) | 0.005 | 0.00 (-0.02, 0.02) | 0.838 |
| 15:0 | PL | 0.02 (0.00, 0.04) | 0.128 | 0.02 (0.00, 0.04) | 0.043 | 0.00 (-0.02, 0.02) | 0.918 |
| 16:0 | CE | 0.26 (-0.29, 0.81) | 0.366 | 0.40 (-0.14, 0.94) | 0.144 | -0.06 (-0.58, 0.47) | 0.842 |
| 16:0 | PL | 1.00 (0.23, 1.78) | 0.011 | 1.06 (0.31, 1.81) | 0.006 | 0.28 (-0.45, 1.01) | 0.466 |
| 16:0 | TG | 1.10 (-1.31, 3.52) | 0.377 | 0.12 (-2.22, 2.47) | 0.924 | 0.10 (-2.19, 2.39) | 0.939 |
| 16:1n7 | CE | 1.46 (0.80, 2.13) | 0.000 | 0.64 (-0.01, 1.29) | 0.051 | 0.40 (-0.23, 1.03) | 0.215 |
| 16:1n7 | PL | 0.24 (0.12, 0.36) | 0.000 | 0.10 (-0.01, 0.22) | 0.076 | 0.04 (-0.07, 0.15) | 0.498 |
| 16:1n7 | TG | 1.31 (0.75, 1.87) | 0.000 | 0.48 (-0.06, 1.03) | 0.081 | 0.06 (-0.47, 0.59) | 0.841 |
| 17:0 | PL | 0.00 (-0.04, 0.03) | 0.828 | 0.02 (-0.02, 0.05) | 0.384 | 0.01 (-0.03, 0.04) | 0.774 |
| 18:0 | CE | -0.14 (-0.29, 0.01) | 0.068 | -0.01 (-0.16, 0.14) | 0.923 | -0.12 (-0.26, 0.03) | 0.117 |
| 18:0 | PL | -0.03 (-0.07, 0.01) | 0.088 | -0.05 (-0.09, -0.02) | 0.005 | 0.00 (-0.03, 0.04) | 0.925 |
| 18:0 | TG | -0.13 (-0.30, 0.04) | 0.121 | -0.09 (-0.25, 0.08) | 0.299 | -0.12 (-0.28, 0.04) | 0.145 |
| 18:1n9 | CE | 2.77 (1.44, 4.09) | 0.000 | 0.42 (-0.87, 1.71) | 0.534 | 0.29 (-0.97, 1.54) | 0.668 |
| 18:1n9 | PL | 1.51 (0.57, 2.46) | 0.002 | 0.43 (-0.49, 1.35) | 0.361 | 0.20 (-0.70, 1.09) | 0.680 |
| 18:1n9 | TG | -0.51 (-3.04, 2.01) | 0.703 | 0.26 (-2.19, 2.72) | 0.845 | -0.67 (-3.07, 1.73) | 0.596 |
| 18:2n6 | CE | -4.87 (-7.25, -2.49) | 0.000 | -1.47 (-3.79, 0.84) | 0.213 | -1.02 (-3.28, 1.24) | 0.383 |

| | | | | | | | |
|---|---|---|---|---|---|---|---|
| 18:2n6 | PL | -2.00 (-3.26, -0.74) | 0.002 | 0.19 (-1.04, 1.41) | 0.777 | -0.36 (-1.55, 0.84) | 0.569 |
| 18:2n6 | TG | -1.15 (-2.93, 0.63) | 0.205 | -0.13 (-1.86, 1.59) | 0.888 | 0.91 (-0.77, 2.60) | 0.292 |
| 18:3n6 | CE | 0.34 (0.06, 0.61) | 0.017 | 0.16 (-0.11, 0.43) | 0.240 | 0.10 (-0.16, 0.37) | 0.451 |
| 18:3n6 | PL | 0.03 (-0.01, 0.07) | 0.149 | 0.02 (-0.02, 0.06) | 0.382 | 0.01 (-0.03, 0.05) | 0.646 |
| 18:3n6 | TG | -0.01 (-0.12, 0.11) | 0.883 | -0.01 (-0.12, 0.10) | 0.887 | 0.04 (-0.07, 0.15) | 0.511 |
| 18:3n3 | CE | 0.03 (-0.12, 0.17) | 0.715 | 0.01 (-0.13, 0.15) | 0.887 | -0.03 (-0.16, 0.11) | 0.702 |
| 18:3n3 | PL | 0.01 (-0.05, 0.07) | 0.763 | 0.02 (-0.04, 0.09) | 0.453 | 0.01 (-0.05, 0.07) | 0.748 |
| 18:3n3 | TG | 0.05 (-0.16, 0.27) | 0.636 | 0.06 (-0.16, 0.27) | 0.620 | 0.09 (-0.12, 0.29) | 0.403 |
| 20:0 | PL | -0.05 (-0.12, 0.01) | 0.118 | 0.02 (-0.04, 0.08) | 0.584 | -0.02 (-0.08, 0.04) | 0.495 |
| 20:3n6 | CE | 0.04 (-0.05, 0.13) | 0.433 | 0.11 (0.02, 0.20) | 0.017 | -0.01 (-0.10, 0.07) | 0.754 |
| 20:3n6 | PL | 0.32 (-0.14, 0.79) | 0.173 | 0.47 (0.02, 0.92) | 0.039 | -0.07 (-0.51, 0.37) | 0.759 |
| 20:3n6 | TG | 0.00 (-0.06, 0.06) | 0.951 | 0.04 (-0.02, 0.10) | 0.195 | 0.01 (-0.05, 0.06) | 0.790 |
| 20:4n6 | CE | -0.18 (-0.83, 0.48) | 0.611 | -0.05 (-0.69, 0.59) | 0.887 | 0.09 (-0.53, 0.71) | 0.787 |
| 20:4n6 | PL | -0.36 (-1.07, 0.35) | 0.324 | -0.34 (-1.03, 0.35) | 0.338 | -0.29 (-0.96, 0.38) | 0.404 |
| 20:4n6 | TG | -0.38 (-0.60, -0.16) | 0.001 | -0.24 (-0.45, -0.03) | 0.027 | 0.00 (-0.21, 0.20) | 0.968 |
| 20:5n3 | CE | 0.05 (-0.56, 0.66) | 0.881 | -0.40 (-1.00, 0.19) | 0.182 | 0.25 (-0.33, 0.83) | 0.413 |
| 20:5n3 | PL | 0.01 (-0.63, 0.65) | 0.977 | -0.48 (-1.10, 0.14) | 0.131 | 0.13 (-0.48, 0.74) | 0.685 |
| 20:5n3 | TG | -0.08 (-0.23, 0.07) | 0.321 | -0.11 (-0.25, 0.04) | 0.150 | 0.03 (-0.11, 0.17) | 0.666 |
| 22:0 | PL | -0.24 (-0.41, -0.08) | 0.004 | -0.14 (-0.30, 0.03) | 0.098 | -0.15 (-0.31, 0.01) | 0.064 |
| 22:5n3 | PL | 0.12 (0.03, 0.21) | 0.013 | 0.03 (-0.06, 0.12) | 0.496 | 0.08 (-0.01, 0.17) | 0.099 |
| 22:5n3 | TG | -0.10 (-0.30, 0.09) | 0.310 | -0.16 (-0.36, 0.03) | 0.097 | -0.10 (-0.28, 0.09) | 0.321 |
| 22:6n3 | CE | -0.03 (-0.19, 0.14) | 0.758 | -0.06 (-0.22, 0.10) | 0.493 | 0.07 (-0.09, 0.22) | 0.421 |
| 22:6n3 | PL | -0.04 (-0.75, 0.66) | 0.914 | -0.60 (-1.28, 0.09) | 0.086 | 0.29 (-0.37, 0.96) | 0.396 |

| | | | | | | | |
|---|---|---|---|---|---|---|---|
| 22:6n3 | TG | -0.09 (-0.41, 0.23) | 0.598 | -0.24 (-0.55, 0.07) | 0.131 | 0.04 (-0.27, 0.34) | 0.816 |
| 24:0 | PL | -0.16 (-0.30, -0.02) | 0.021 | -0.13 (-0.27, 0.00) | 0.048 | -0.13 (-0.26, 0.00) | 0.044 |
| SCD1 | CE | 0.12 (0.07, 0.17) | 0.000 | 0.05 (0.00, 0.10) | 0.067 | 0.04 (-0.01, 0.09) | 0.146 |
| SCD1 | PL | 0.01 (0.00, 0.01) | 0.000 | 0.00 (-0.00, 0.01) | 0.126 | 0.00 (-0.00, 0.00) | 0.453 |
| SCD1 | TG | 0.04 (0.03, 0.06) | 0.000 | 0.02 (0.00, 0.03) | 0.049 | 0.00 (-0.01, 0.02) | 0.884 |

Data are results from the analysis of the mixed effects model, with each individual fatty acid as the dependent variable; individual as a random intercept; group, time, weight, sex, and age as fixed effects, and an interaction term of group and time. Values are means (95% lower and upper limits) estimate of the change in proportions of plasma fatty acids in the lipid fractions between the baseline and after the interventional period compared to water group.

**Supplemental Table 3. Pearson's correlation between change in liver fat and change in fatty acids over 24 weeks**

| Fatty acid | Fraction | Water ($n$=9) | | Sugar-sweetened Soda ($n$=8) | | semi-skimmed Milk ($n$=8) | | Aspartame-sweetened Soda ($n$=11) | | Total ($n$=36) | |
|---|---|---|---|---|---|---|---|---|---|---|---|
| | | *r* | *p* | *r* | *p* | *r* | *p* | *r* | *p* | *r* | *p* |
| 14:0 | CE | -0.132 | 0.735 | 0.65 | 0.081 | 0.237 | 0.572 | 0.294 | 0.38 | 0.352 | 0.035 |
| 14:0 | PL | 0.142 | 0.715 | 0.477 | 0.232 | -0.039 | 0.926 | 0.158 | 0.642 | 0.243 | 0.154 |
| 14:0 | TG | 0.543 | 0.131 | 0.526 | 0.181 | -0.235 | 0.575 | 0.182 | 0.593 | 0.296 | 0.08 |
| 15:0 | CE | -0.606 | 0.084 | 0.589 | 0.124 | -0.321 | 0.438 | -0.055 | 0.873 | -0.012 | 0.942 |
| 15:0 | PL | -0.512 | 0.159 | 0.261 | 0.533 | -0.383 | 0.348 | 0.242 | 0.473 | 0.061 | 0.724 |
| 16:0 | CE | 0.35 | 0.356 | 0.367 | 0.371 | -0.524 | 0.183 | -0.133 | 0.696 | 0.163 | 0.342 |
| 16:0 | PL | -0.485 | 0.186 | 0.054 | 0.898 | -0.405 | 0.32 | -0.037 | 0.915 | 0.008 | 0.961 |
| 16:0 | TG | 0.601 | 0.087 | 0.791 | 0.019 | -0.052 | 0.902 | 0.422 | 0.196 | 0.504 | 0.002 |
| 16:1n7 | CE | 0.679 | 0.044 | 0.176 | 0.676 | 0.523 | 0.184 | 0.245 | 0.469 | 0.511 | 0.001 |
| 16:1n7 | PL | 0.859 | 0.003 | 0.107 | 0.8 | 0.301 | 0.468 | -0.167 | 0.623 | 0.449 | 0.006 |
| 16:1n7 | TG | 0.831 | 0.005 | 0.26 | 0.535 | -0.01 | 0.982 | -0.293 | 0.382 | 0.387 | 0.02 |
| 17:0 | PL | -0.617 | 0.077 | -0.194 | 0.646 | -0.685 | 0.061 | -0.15 | 0.66 | -0.374 | 0.025 |
| 18:0 | CE | 0.01 | 0.979 | 0.671 | 0.069 | -0.045 | 0.916 | -0.631 | 0.037 | -0.187 | 0.275 |
| 18:0 | PL | 0.679 | 0.044 | -0.008 | 0.984 | 0.638 | 0.088 | -0.007 | 0.983 | 0.087 | 0.612 |
| 18:0 | TG | 0.476 | 0.196 | 0.366 | 0.373 | 0.288 | 0.49 | 0.358 | 0.279 | 0.31 | 0.066 |
| 18:1n9 | CE | 0.32 | 0.401 | 0.199 | 0.636 | -0.016 | 0.97 | 0.248 | 0.462 | 0.352 | 0.035 |

| | | | | | | | | | | | |
|---|---|---|---|---|---|---|---|---|---|---|---|
| 18:1n9 | PL | 0.716 | 0.03 | 0.313 | 0.45 | -0.103 | 0.807 | -0.1 | 0.771 | 0.339 | 0.043 |
| 18:1n9 | TG | -0.618 | 0.076 | -0.654 | 0.079 | 0.135 | 0.75 | -0.215 | 0.525 | -0.383 | 0.021 |
| 18:2n6 | CE | -0.517 | 0.154 | -0.307 | 0.46 | -0.126 | 0.766 | -0.216 | 0.524 | -0.423 | 0.01 |
| 18:2n6 | PL | -0.344 | 0.364 | -0.166 | 0.695 | -0.072 | 0.865 | -0.213 | 0.529 | -0.318 | 0.058 |
| 18:2n6 | TG | -0.578 | 0.103 | -0.335 | 0.417 | -0.047 | 0.912 | -0.59 | 0.056 | -0.46 | 0.005 |
| 18:3n6 | CE | 0.557 | 0.119 | 0.006 | 0.989 | 0.313 | 0.45 | 0.354 | 0.286 | 0.383 | 0.021 |
| 18:3n6 | PL | 0.85 | 0.004 | 0.31 | 0.454 | -0.043 | 0.92 | 0.037 | 0.915 | 0.354 | 0.034 |
| 18:3n6 | TG | 0.312 | 0.414 | 0.23 | 0.584 | -0.496 | 0.211 | -0.206 | 0.543 | 0.012 | 0.946 |
| 18:3n3 | CE | 0.143 | 0.714 | 0.102 | 0.811 | -0.048 | 0.911 | 0.362 | 0.274 | 0.166 | 0.332 |
| 18:3n3 | PL | 0.599 | 0.088 | -0.009 | 0.983 | 0.059 | 0.89 | 0.122 | 0.721 | 0.202 | 0.238 |
| 18:3n3 | TG | -0.055 | 0.887 | -0.298 | 0.474 | -0.038 | 0.929 | -0.144 | 0.673 | -0.11 | 0.524 |
| 20:0 | PL | -0.551 | 0.124 | 0.554 | 0.154 | 0.429 | 0.289 | -0.312 | 0.35 | -0.159 | 0.355 |
| 20:3n6 | CE | 0.382 | 0.311 | 0.274 | 0.511 | -0.01 | 0.98 | 0.152 | 0.655 | 0.232 | 0.172 |
| 20:3n6 | PL | -0.005 | 0.989 | -0.146 | 0.731 | 0.059 | 0.89 | -0.052 | 0.879 | 0.052 | 0.763 |
| 20:3n6 | TG | 0.179 | 0.645 | -0.467 | 0.243 | 0.206 | 0.624 | -0.545 | 0.083 | -0.128 | 0.455 |
| 20:4n6 | CE | 0.051 | 0.895 | -0.07 | 0.87 | -0.065 | 0.879 | -0.27 | 0.422 | -0.161 | 0.348 |
| 20:4n6 | PL | -0.1 | 0.798 | 0.048 | 0.91 | 0.084 | 0.843 | -0.386 | 0.241 | -0.143 | 0.406 |
| 20:4n6 | TG | 0.532 | 0.141 | 0.13 | 0.758 | 0.133 | 0.754 | -0.167 | 0.624 | -0.02 | 0.906 |
| 20:5n3 | CE | -0.376 | 0.318 | -0.253 | 0.545 | -0.002 | 0.997 | 0.424 | 0.194 | 0.03 | 0.864 |
| 20:5n3 | PL | -0.499 | 0.172 | 0.453 | 0.26 | 0.608 | 0.11 | 0.63 | 0.038 | 0.072 | 0.674 |
| 20:5n3 | TG | 0.337 | 0.375 | -0.389 | 0.341 | 0.185 | 0.662 | 0.435 | 0.182 | 0.124 | 0.473 |
| 22:0 | PL | -0.342 | 0.368 | -0.468 | 0.242 | 0.07 | 0.869 | 0.555 | 0.077 | 0.098 | 0.568 |
| 22:5n3 | PL | -0.512 | 0.159 | 0.422 | 0.298 | 0.558 | 0.151 | 0.564 | 0.071 | 0.02 | 0.91 |

| | | | | | | | | | | | |
|---|---|---|---|---|---|---|---|---|---|---|---|
| 22:5n3 | TG | -0.339 | 0.372 | -0.671 | 0.069 | 0.107 | 0.8 | 0.148 | 0.664 | -0.205 | 0.23 |
| 22:6n3 | CE | -0.044 | 0.911 | 0.214 | 0.611 | -0.274 | 0.511 | -0.116 | 0.734 | -0.13 | 0.45 |
| 22:6n3 | PL | 0.483 | 0.188 | 0.087 | 0.837 | -0.075 | 0.861 | 0.146 | 0.668 | 0.236 | 0.166 |
| 22:6n3 | TG | -0.07 | 0.857 | -0.353 | 0.391 | 0.158 | 0.708 | 0.362 | 0.275 | 0.028 | 0.871 |
| 24:0 | PL | -0.395 | 0.293 | -0.21 | 0.617 | -0.111 | 0.794 | 0.085 | 0.805 | -0.116 | 0.502 |
| SCD1 | CE | 0.652 | 0.057 | 0.112 | 0.791 | 0.577 | 0.134 | 0.305 | 0.363 | 0.511 | 0.001 |
| SCD1 | PL | 0.867 | 0.002 | 0.118 | 0.781 | 0.342 | 0.407 | -0.149 | 0.661 | 0.468 | 0.004 |
| SCD1 | TG | 0.42 | 0.261 | -0.222 | 0.598 | 0.007 | 0.986 | -0.499 | 0.118 | 0.135 | 0.434 |

Log-transformed data of liver fat were utilized to compute Pearson’s correlation between the change in liver fat and the change in each fatty acid over 24 weeks of intake of test beverages. The *p*-value column illustrates the significance level by Pearson’s correlation.
